# The Effects of Pain on Brain and Behavior During Cognitive and Motor Performances in Older Adults

**DOI:** 10.1093/geroni/igaa057.924

**Published:** 2020-12-16

**Authors:** Hannah Pakray, Elizabeth Seng, Meltem Izzetoglu, Roee Holtzer

**Affiliations:** 1 Yeshiva University, Bronx, New York, United States; 2 Villanova University, Villanova, Pennsylvania, United States

## Abstract

Pain is prevalent and associated with adverse outcomes in older adults. The moderating effect of pain on cortical control of locomotion has not been assessed. This study examined the effects of subjective pain on changes in fNIRS-derived oxygenated hemoglobin (HbO2), gait velocity, and cognitive accuracy from single to dual-task walking conditions among older adults. Participants (n=383; 55% female; mean age=76) were community-residing older adults. Participants completed two single tasks [Single-Task-Walk (STW) and Cognitive Interference (Alpha)] and the Dual-Task-Walk (DTW), during which participants performed the two single tasks simultaneously. The MOS- PSS and PES were used to assess pain severity and interference. Gait velocity was assessed with an electronic gait mat, cognitive accuracy was measured using the rate of correct letter generation. Linear mixed effects models revealed that HbO2 increased in DTW compared to STW (p < .001) and Alpha (p < .05). The presence of perceived pain was associated with an attenuated increase in HbO2 from Alpha to DTW (p < .05). Amongst those with pain, worse pain severity was associated with an attenuated increase in HbO2 from STW to DTW (p < .001). Pain interference did not moderate the increase in HbO2 from single to dual tasks. Pain did not have a moderating effect on behavioral outcomes. These results indicate that task-related changes in the hemodynamic response in the prefrontal cortex during walking may be a sensitive marker to the effects of subjective pain on brain function in older adults.

